# Genetic alterations and their clinical implications in gastric cancer peritoneal carcinomatosis revealed by whole-exome sequencing of malignant ascites

**DOI:** 10.18632/oncotarget.6977

**Published:** 2016-01-22

**Authors:** Byungho Lim, Chan Kim, Jeong-Hwan Kim, Woo Sun Kwon, Won Seok Lee, Jeong Min Kim, Jun Yong Park, Hyo Song Kim, Kyu Hyun Park, Tae Soo Kim, Jong-Lyul Park, Hyun Cheol Chung, Sun Young Rha, Seon-Young Kim

**Affiliations:** ^1^ Genome Structure Research Center, Korea Research Institute of Bioscience and Biotechnology (KRIBB), Daejeon, Korea; ^2^ Division of Medical Oncology, Department of Internal Medicine, Yonsei Cancer Center, Yonsei University College of Medicine, Seoul, Korea; ^3^ Song-Dang Institute for Cancer Research, Yonsei University College of Medicine, Seoul, Korea; ^4^ Epigenomics Research Center, Korea Research Institute of Bioscience and Biotechnology (KRIBB), Daejeon, Korea; ^5^ Department of Internal Medicine and Institute of Gastroenterology, Yonsei University College of Medicine, Seoul, Korea; ^6^ Brain Korea 21 Project for Medical Science, Yonsei University College of Medicine, Seoul, Korea; ^7^ Department of Functional Genomics, University of Science and Technology, Daejeon, Korea

**Keywords:** exome sequencing, gastric cancer, malignant ascites, peritoneal carcinomatosis, somatic mutation

## Abstract

Peritoneal carcinomatosis accompanied by malignant ascites is a major cause of death of advanced gastric cancer (GC). To comprehensively characterize the underlying genomic events involved in GC peritoneal carcinomatosis, we analyzed whole-exome sequences of normal gastric tissues, primary tumors, and malignant ascites from eight GC patients. We identified a unique mutational signature biased toward C-to-A substitutions in malignant ascites. In contrast, the patients who received treatment of adjuvant chemotherapy showed a high rate of C-to-T substitutions along with hypermutation in malignant ascites. Comparative analysis revealed several candidate mutations for GC peritoneal carcinomatosis: recurrent mutations in *COL4A6*, *INTS2*, and *PTPN13*; mutations in druggable genes including *TEP1, PRKCD, BRAF, ERBB4, PIK3CA, HDAC9, FYN, FASN, BIRC2, FLT3, ROCK1, CD22*, and *PIK3C2B*; and mutations in metastasis-associated genes including *TNFSF12, L1CAM, DIAPH3, ROCK1, TGFBR1, MYO9B, NR4A1*, and *RHOA*. Notably, gene ontology analysis revealed the significant enrichment of mutations in the Rho-ROCK signaling pathway-associated biological processes in malignant ascites. At least four of the eight patients acquired somatic mutations in the Rho-ROCK pathway components, suggesting the possible relevance of this pathway to GC peritoneal carcinomatosis. These results provide a genome-wide molecular understanding of GC peritoneal carcinomatosis and its clinical implications, thereby facilitating the development of effective therapeutics.

## INTRODUCTION

To metastasize to distant organs, cancer cells exploit three major routes of dissemination: blood vessels, lymphatic vessels, and direct seeding into the body cavity [1]. In metastatic GC, direct seeding into the peritoneal cavity occurs in more than 55~60% of patients. The resulting peritoneal carcinomatosis is the most common and important clinical manifestation, leading to a poor prognosis [2, 3]. Once peritoneal dissemination takes place, it is regarded as incurable; it is seldom responsive to surgical resection and responds poorly to conventional chemotherapy regimens, which cannot cross the blood-peritoneal barrier [4]. Therefore, there is an unmet clinical need for the development of an effective therapy for peritoneal carcinomatosis, and it is important to comprehensively understand the molecular mechanisms involved in the peritoneal dissemination of GC cells.

Peritoneal carcinomatosis of GC is a complex and dynamic process involving multiple steps [5–7]. First, GC cells detach from a primary serosa-invasive tumor mass through the down-regulation of intercellular adhesion molecules, such as E-cadherin, and they then gain access to the peritoneal cavity [6]. Next, the free GC cells adhere to the distant mesothelium, the innermost monolayer of the peritoneum, through adhesion molecules such as CD44 and selectins. After this attachment, the GC cells invade the subperitoneal connective tissue under the guidance of motility factors and matrix metalloproteinases [5]. Later, GC cells activate angiogenesis by secreting VEGF, and they proliferate to develop seeding nodules through the regulation of autocrine and paracrine loops involving growth factors and chemokines produced by GC cells and stromal cells [6].

It is currently well recognized that the accumulation of genetic alterations and the subsequent acquisition of phenotypic traits is responsible for the metastasis of various cancers [8, 9]. However, because most studies have focused on cellular and phenotypic changes involved in peritoneal metastasis, the genetic alterations responsible for the acquisition of these phenotypic traits have been poorly elucidated.

Recent advances in next-generation sequencing have made whole-exome sequencing not only cost-effective but also technically feasible [10]. In particular, it is now possible to perform a genome-wide analysis of peritoneal carcinomatosis with small amounts of DNA obtained from malignant ascites, which is the most easily obtainable sample for metastatic GC by a simple paracentesis.

In this study, we aimed to identify genomic alterations of GC peritoneal carcinomatosis. To achieve this goal, we performed whole-exome sequencing using normal gastric tissues, primary tumors, and malignant ascites samples from eight GC patients with peritoneal carcinomatosis.

## RESULTS

### Clinicopathologic characteristics of GC patients with peritoneal carcinomatosis

**Figure 1:**
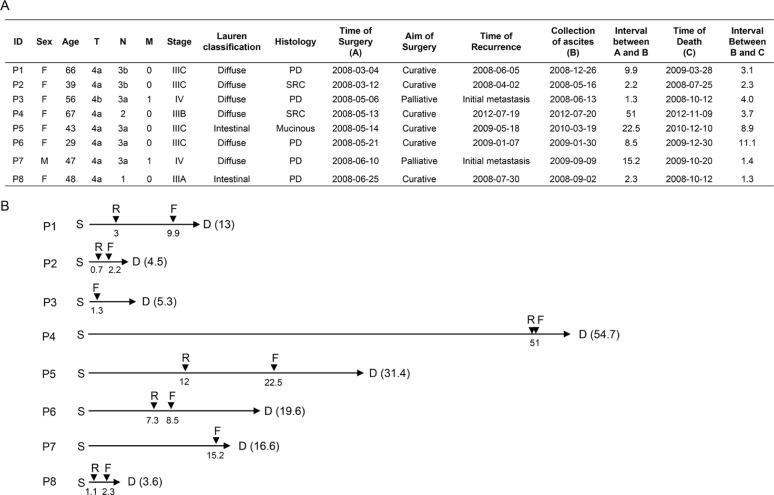
Clinical information (**A**) Clinicopathologic characteristics of eight GC patients with peritoneal carcinomatosis. PD, poorly-differentiated; SRC, signet ring cell. (**B**) Clinical time course of eight GC patients with peritoneal carcinomatosis. Numbers indicate the time interval (in months) from the time of surgery (S). D, time of death; F, time of ascites fluid collection; R, time of recurrence.

Six of eight patients were diagnosed as diffuse-type GC with histological features that are consistent with poorly differentiated adenocarcinoma, whereas two patients were intestinal-type GC (Figure 1A). All patients had an advanced disease stage at the initial presentation (stage III or IV in AJCC 7th staging system) and received curative or palliative gastrectomy (Figure 1A). At the time when their diseases became metastatic or recurrent, all patients had clinically distinct peritoneal carcinomatosis with cytologically confirmed malignant ascites (Supplementary Figure 1). The median time from surgery to recurrence was 3 months (range 0.7–50.9 months), and the median overall survival duration was 13.4 months (range 3.6–54.7 months) (Figure 1B).

### Mutation patterns identified from primary tumors and malignant ascites

To explore somatic mutations involved in GC peritoneal carcinomatosis, we analyzed whole-exome sequences of 24 tissue samples that consist of triplets of normal gastric tissues, primary tumors, and malignant ascites from eight GC patients. Mutations identified are listed in Supplementary Table 1 and 2. When tumor purity was mathematically estimated using ASCAT v2.1, the average tumor purity was 24.0% in primary tumors and 45.5% in malignant ascites. The highly infiltrative feature of advanced diffuse-type GC may result in the low tumor purity, especially in primary tumors [11]. Our observation that the number of mutations in primary tumors significantly correlated with the average VAF (Supplementary Figure 2, *r* = 0.74, *P* = 0.037) may indicate that our mutation analysis may, in part, be affected by low tumor purity.

**Figure 2:**
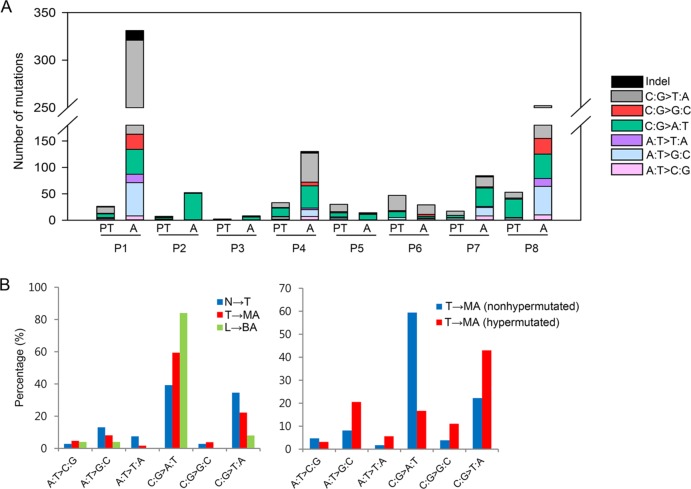
Statistical analyses of somatic mutations (**A**) The number and base substitutions of mutations. PT, primary tumor; A, malignant ascites (**B**) The proportion of base substitutions found in primary tumors and ascites. N, normal tissues; T, primary tumors; MA, malignant ascites; L, normal blood; BA, benign ascites.

The average sequencing depth of mutations in the protein-coding regions was ~61.1× in primary tumors and ~63.9× in malignant ascites. The average number of mutations per patient was ~27 in primary tumors and ~113 in malignant ascites (Figure 2A). In particular, two patients P1 and P8 had hypermutation in malignant ascites, but not in primary tumors (Figure 2A). However, malignant ascites samples from the two patients were negative for a microsatellite instability test (Supplementary Figure 3). Instead, the hypermutation phenotype is most likely associated with chemotherapy. The two hypermutated patients received chemotherapy (FL (5-FU and leucovorin)/Docetaxel for P1 and FP (5-FU and cisplatin) for P8) before the collection of malignant peritoneal fluids, whereas other patients who do not have hypermutation had the treatment after the collection of malignant ascites. This result is consistent with a previous report that tumors treated with the chemotherapeutic drug temozolomide were hypermutated [12].

To dissect a mutational signature of primary tumors and malignant ascites, we examined the spectrum of base substitutions. The analysis revealed an unusual high proportion of C-to-A transversions during the progression from primary tumors to malignant ascites, with the exception of the two hypermutated patients (P1 and P8) (Figure 2A and 2B). On average, C-to-A transversions accounted for ~39.3% in primary tumors, and ~59.4% (except for hypermutated patients) in malignant ascites (Figure 2B, left panel).

To assess whether the C-to-A transversion is a general mutational signature that occurs in ascites, we additionally performed whole-exome sequencing of benign ascites derived from three liver cirrhosis patients. Despite the non-cancerous characteristics of the samples, a small number of somatic mutations were identified in the three liver cirrhosis-derived ascites: 10 mutations in patient N1; no mutations in patient N2; and 15 mutations in patient N3 (Supplementary Table 3). Although we cannot rule out the possibility that the small number of mutations may be background errors, the proportion of C-to-A transversions in liver cirrhosis-derived benign ascites was as high as 84% (21 of 25 mutations) (Figure 2B, left panel).

Next, we compared distinct mutational patterns between hypermutated (P1 and P8) and the other non-hypermutated samples. Hypermutated malignant ascites exhibited a higher proportion of C-to-T transitions (43.0%) and a lower proportion of C-to-A transversions (16.7%) compared to non-hypermutated malignant ascites (22.2% C-to-T transitions and 59.4% C-to-A transversions) (Figure 2B, right panel). In addition, the examination of 96 base substitutions displayed a distinct base substitution signature: non-hypermutated malignant ascites exhibited a biased base substitution pattern toward C[C→A]G, C[C→A]C, C[C→A]A, C[C→A]T, T[C→A]G, and T[C→A]A tri-nucleotide contexts (Supplementary Figure 4).

### Somatic mutations and their clinical associations

Mutations relevant to a metastatic process may be positively selected and accumulated during metastasis in a time-dependent manner [13]. Therefore, we hypothesized that a longer time interval from gastrectomy to peritoneal carcinomatosis would increase the chance for numerous mutations to be accumulated in malignant ascites. To test this hypothesis, we assessed the correlation between the number of mutations and the elapsed time from gastrectomy to the development of peritoneal carcinomatosis (the length between S and F in Figure 1B). Two patients with hypermutation were excluded from this analysis because they may have distinct mutagenic processes from non-hypermutated patients as shown in Figure 2B. We observed a tendency toward a positive correlation between the number of mutations in malignant ascites and the time interval from gastrectomy to the development of peritoneal carcinomatosis (Supplementary Figure 5A, *r* = 0.74, *P* = 0.086). We also hypothesized that the population of tumor cells harboring mutations may expand in malignant ascites due to the growth advantage provided by mutations, thereby resulting in a time-dependent increase of mutational VAFs in malignant ascites. We observed a significant positive correlation between mutational VAFs in malignant ascites and the elapsed time from gastrectomy to the development of peritoneal carcinomatosis (Supplementary Figure 5B, *r* = 0.79, *P* = 0.045). However, these correlation patterns were not observed with mutation profiles of primary tumors (Supplementary Figure 5C and 5D)

Some mutations found in malignant ascites would be associated with poor disease prognosis because tumor cells harboring these mutations overcame multiple metastatic barriers. Therefore, we tested whether the increased number and VAFs of mutations in malignant ascites correlate with survival duration after peritoneal carcinomatosis (the length between F and D in Figure 1B). The number and VAFs of mutations in the malignant ascites showed a mild negative correlation trend with overall survival duration after peritoneal carcinomatosis (Supplementary Figure 5E and 5F). These negative correlation trends were not observed with mutation profiles of primary tumors (Supplementary Figure 5G and 5H).

### Biological processes targeted by mutations

**Figure 3:**
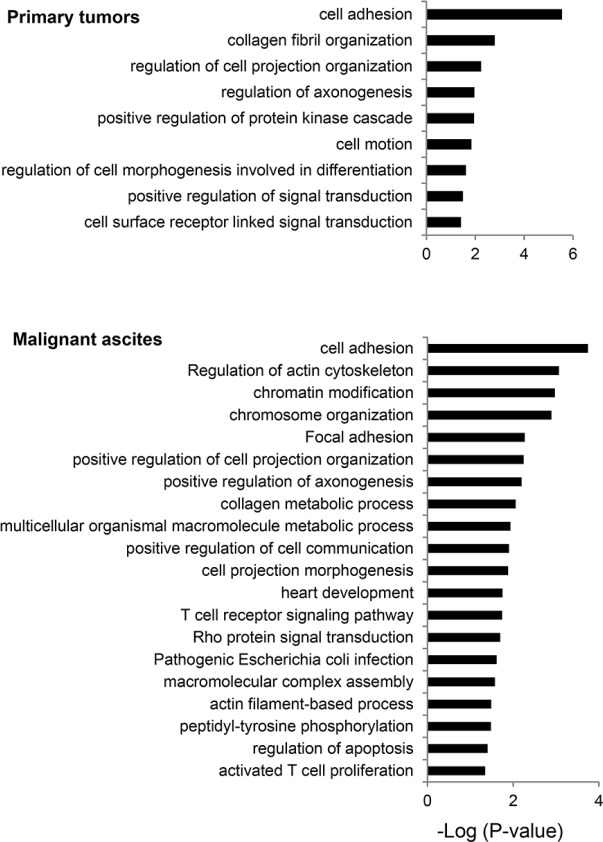
Biological processes frequently mutated in primary tumors and malignant ascites The functional terms significantly overrepresented are presented as −log_10_ (*P*-value).

We examined biological processes frequently mutated in primary tumors and malignant ascites. Consistent with a previous report [14], genes involved in cell adhesion and chromosome organization were frequently mutated in our GC patients as well. In primary tumors, genes involved in cell adhesion, collagen organization, axonogenesis, cell motility, and cell morphogenesis were frequently mutated (Figure 3). In malignant ascites, functional terms, such as actin cytoskeleton, chromosome organization, focal adhesion, Rho-protein signaling, immune activation, and apoptosis, were significantly overrepresented to be biological processes affected by mutations (Figure 3). The enrichment of the Rho signaling pathway-associated functional terms, such as actin cytoskeleton, focal adhesion, and Rho-protein signaling, may suggest the importance of the Rho signaling pathway in GC peritoneal carcinomatosis.

### Clonality of primary tumors and malignant ascites

**Figure 4:**
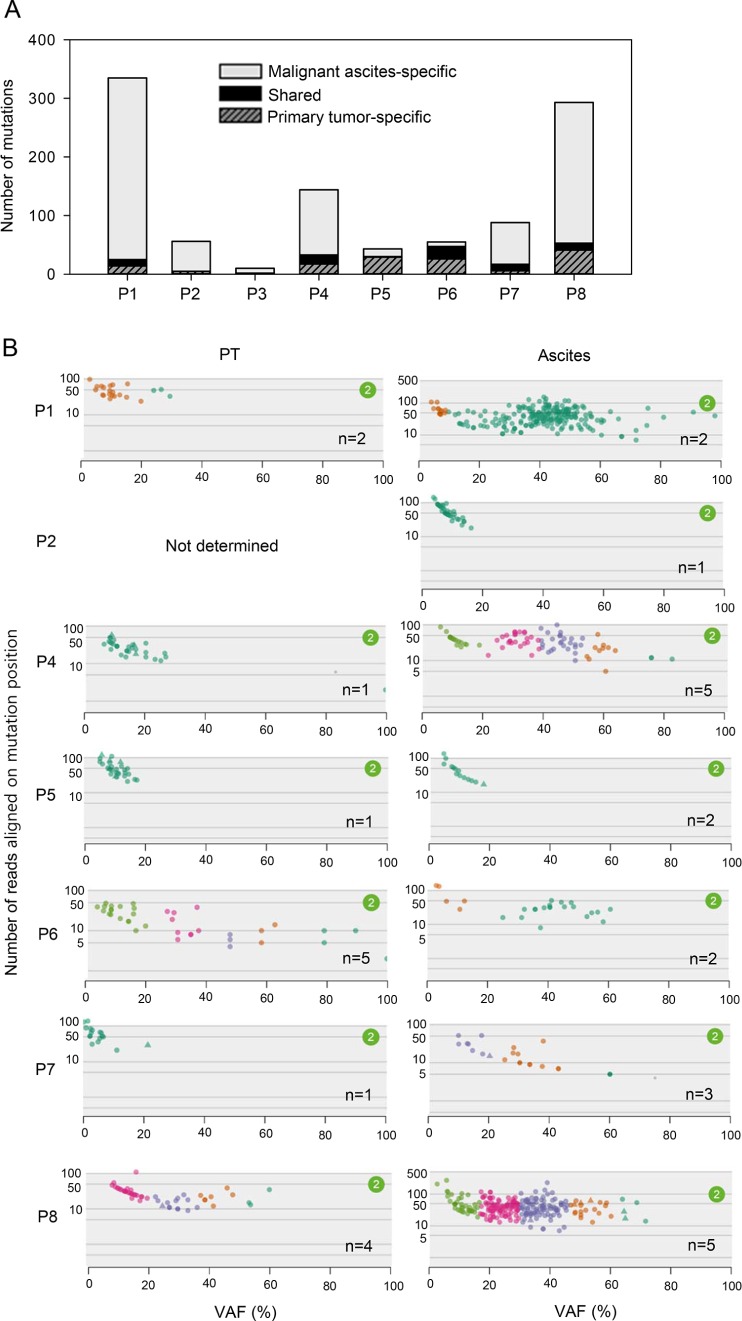
Clonality of primary tumors and malignant ascites (**A**) The number of shared (between primary tumors and malignant ascites) and tumor type-specific mutations. (**B**) Clonality of primary tumors and malignant ascites analyzed by SciClone. Number (*n*) indicates the number of clonal clusters.

A recent study revealed that primary tumors and the matched metastases share a majority of mutations [15], exhibiting a high mutational concordance between them. However, since malignant ascites from our patients were formed after curative or palliative gastrectomy followed by recurrence (Figure 1A), it is unlikely that our primary tumors and the matched malignant ascites would exhibit a high mutational concordance. In fact, the genomic concordance between them was relatively low compared to the previous synchronous metastasis study (Figure 4A, range 0–38%). Instead, malignant ascites-specific mutations accounted for the highest proportion in our tumor samples (Figure 4A, range 15–93%).

To infer the clonality of primary tumors and malignant ascites, we performed SciClone analysis. Two patients, P2 and P3, were excluded from this analysis because these patients had too small number of mutations to cluster. Consistent with the low genomic concordance, clonality patterns between primary tumors and malignant ascites were largely different (Figure 4B). Some patients exhibited an increased clonality during metastasis, whereas the others showed decreased or similar clonality (Figure 4B), revealing the heterogeneous clonal evolution during metastasis. Patients (P4 and P7), who exhibited increased clonality during metastasis, showed low genomic concordance between primary tumors and malignant ascites, whereas a patient P6, who exhibited decreased clonality during metastasis showed the relatively high genomic concordance (Figure 4A and 4B). This pattern is consistent with a recent report that the genomic concordance between primary tumors and metastases is associated with clonality change [16]. Accordingly, these results suggest that gastrectomy followed by recurrence may alter the clonal composition of the tumors, although we cannot completely rule out the issue of low tumor purity in primary tumors.

Next, we examined the clinical implication of tumor clonality. A previous study showed that patients exhibiting high clonality had poor prognosis in GC [17]. Consistent with this observation, higher clonality in primary tumors showed a trend of faster development of peritoneal calcinomatosis after gastrectomy (Supplementary Figure 6A, *r* = −0.62). Higher clonality in malignant ascites exhibited a trend toward poorer overall survival after peritoneal carcinomatosis (Supplementary Figure 6B, *r* = −0.44). In addition, increased clonality during metastasis positively correlated with longer time interval from gastrectomy to peritoneal carcinomatosis (Supplementary Figure 6C, *r* = 0.68) and negatively correlated with overall survival after peritoneal carcinomatosis (Supplementary Figure 6D, *r* = −0.71).

### Druggable genes mutated in each patient

**Figure 5:**
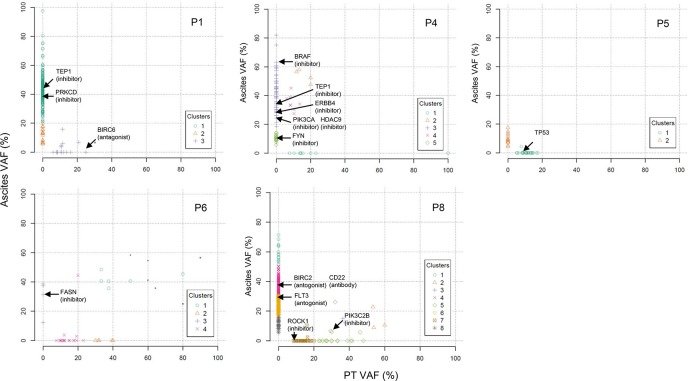
Two dimensional clustering of mutations presented as primary tumors versus malignant ascites Druggable genes are indicated by arrows on the basis of the DGIdb.

We performed two-dimensional clustering of mutations using SciClone (Figure 5). To provide therapeutic options for each patient, we investigated whether tumor clones analyzed by clustering are therapeutically targetable. We employed Drug-Gene interaction database (DGIdb) to search for druggable genes. In patient P1, two clonal clusters emerged in malignant ascites (Figure 5). One of the two emerging clones may be targetable by *TEP1* (e.g. GRN163L) or *PRKCD* (e.g. KAI-9803) inhibitors (Figure 5 and Supplementary Table 4). In patient P4, two of four malignant ascites-emerging clones may be targetable by inhibitors against *BRAF* (e.g. Vemurafenib), *TEP1*, *ERBB4*, *PIK3CA*, *HDAC9*, or *FYN* (Figure 5 and Supplementary Table 4). In patient P6, *FASN* may be a druggable target for a malignant ascites-emerging clone (Figure 5 and Supplementary Table 4). In patient P8, inhibitors against *BIRC2* or *FLT3*, or a monoclonal antibody against CD22 may be potential drugs for two malignant ascites-emerging clones (Figure 5 and Supplementary Table 4). However, we predict that the patient 8 may be resistant to the drug treatment because of the highly heterogeneous clonality.

### Somatic mutations found in GC peritoneal carcinomatosis

**Figure 6:**
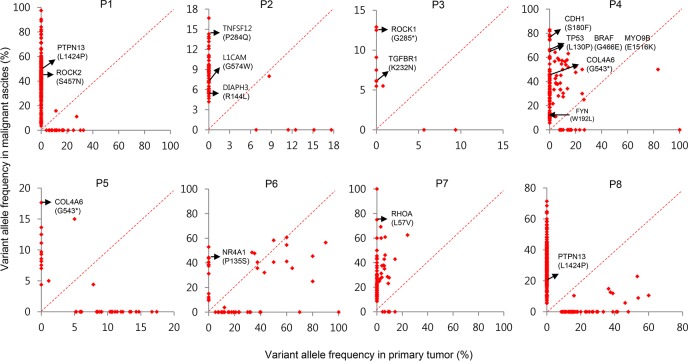
Paired VAFs of mutations in primary tumors and malignant ascites Scatter plots presented as VAFs in malignant ascites versus VAFs in primary tumors. Metastasis-associated mutations are indicated by arrows.

**Figure 7:**
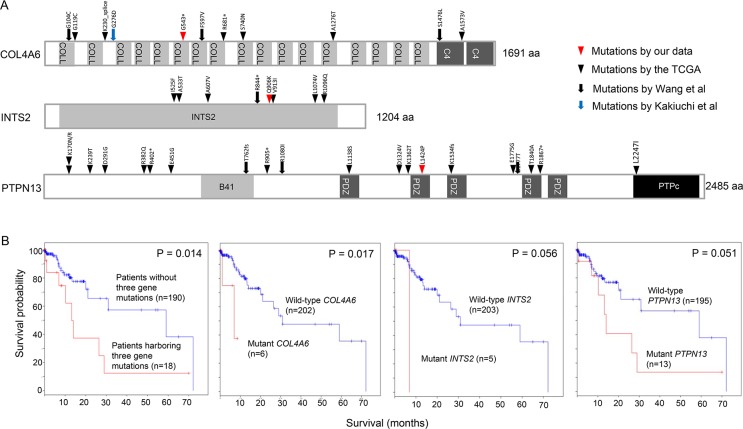
Recurrent mutations in *COL4A6*, *INTS2*, and *PTPN13* (**A**) The positions of *COL4A6*, *INTS2*, and *PTPN13* mutations found in four different GC studies. (**B**) Kaplan-Meier survival curves analyzed by the absence or presence of *COL4A6*, *INTS2*, and *PTPN13* mutations in the TCGA GC dataset. The log rank *P*-value is shown.

We found numerous mutations in GC peritoneal carcinomatosis (Supplementary Tables 1 and 2) and successfully confirmed a few of them by Sanger sequencing (Supplementary Figures 7 and 8). In particular, we identified three recurrent mutations that occurred at the same genomic position in two malignant ascites samples. A *COL4A6* mutation was identified at genomic position 107431221 on the X chromosome in patients P4 and P5 (Figure 6 and Supplementary Figure 8). This mutation is a nonsense mutation (G543*) that introduces a premature stop codon at amino acid 543 of COL4A6 (Figure 7A). Because this mutation potentially truncates ~68% of the COL4A6, including the collagen helix and C4 domains (Figure 7A), it may lead to the functional loss of COL4A6. *COL4A6* mutations were identified in 3% of the TCGA GC patients (6 of 220 patients). A second recurrent mutation was an *INTS2* mutation that was located at genomic position 59949712 on chromosome 17 in patients P2 and P4 (Supplementary Figure 8). This mutation is a missense mutation that changes a glutamine at amino acid 906 to a lysine (Figure 7A). In total, 3% of the TCGA GC patients (6 of 220 patients) acquired *INTS2* mutations. Lastly, a *PTPN13* mutation was identified at genomic position 87687597 on chromosome 4 in patients P1 and P8 (Figure 6 and Supplementary Figure 8). This mutation is a missense mutation (L1424P) located in a protein-interacting PDZ domain (Figure 7A) and has a high probability of being functionally deleterious, as predicted by Mutation Assessor (prediction score = 3.74) [18]. *PTPN13* mutations were found in 6% of the TCGA GC patients (12 of 220 patients). Notably, GC patients harboring *COL4A6*, *INTS2*, or *PTPN13* mutations exhibited a worse survival probability than patients lacking mutations in the genes in the TCGA GC cohort (Figure 7B).

In addition, at least four of eight malignant ascites samples acquired mutations in the Rho-ROCK pathway components. In patient P7, we identified a *RHOA* mutation encoding the L57V substitution (Figure 6). The RHOA L57V mutation was recently identified to be a hotspot mutation for diffuse-type GC [13, 19, 20]. In patient P3, we identified a *ROCK1* nonsense mutation (Figure 6). This mutation introduces a premature stop codon at the 285th amino acid of ROCK1, leading to the truncation of ~79% of ROCK1. The truncated portion includes a part of the kinase domain (aa 76–338), the HR1 Rho-binding repeat (aa 458–537), the entire Rho-binding domain (aa 948–1014), and the PH domain (aa 1119–1317). The lack of a Rho-binding domain by the *ROCK1* mutation could impair the Rho-ROCK pathway similarly to the *RHOA* L57V mutation. In patient P4, we identified a missense mutation (E1516K) in *MYO9B* (Figure 6), a RhoGAP with inhibitory activity against RhoA [21]. This mutation is predicted to be deleterious by Mutation Assessor (prediction score = 2.28). A mutation (W192L, prediction score = 2.19) in *FYN*, a regulator of RhoA activity [22], was also identified in patient P4 (Figure 6). We also identified a *ROCK2* mutation (S457N) in patient P1 (Figure 6). Accordingly, these results may suggest the importance of the Rho-ROCK pathway in peritoneal carcinomatosis.

We next examined mutations found in metastasis-associated genes. In patient P2, we identified a mutation in *TNFSF12* (Figure 6, P284Q, prediction score = 1.85) that plays a role in metastasis by upregulating VEGF through the NF-κB signaling pathway [23]. We also identified a mutation in *L1CAM* in patient P2 (Figure 6, G574W, prediction score = 2.47) that was demonstrated to be a relevant metastatic mediator targeted by plasmin [24]. A *DIAPH3* mutation (Figure 6, R144L, prediction score = 1.91) was also identified in patient P2. *DIAPH3* plays roles in cancer cell invasion, amoeboid cell behavior, and metastasis *in vivo* [25]. As predicted by Mutation Assessor, all these mutations have a high probability of being functionally deleterious.

We also found two mutations in the TGF-β pathway genes. One was a *TGFBR1* mutation (Figure 6, K232N, prediction score = 5.08) in patient P3, and the other was a mutation in *NR4A1* (Figure 6, P135S, prediction score = 1.55) in patient P6, which activates metastatic progression in a TGF-β-dependent manner [26].

## DISCUSSION

Here, we performed whole-exome sequencing to identify somatic mutations involved in GC peritoneal carcinomatosis. Recently published genomic analyses focused entirely on the primary tumor mass [13, 27, 28]. These studies provided limited information regarding the metastatic disease after recurrence. To address this issue, we used intraperitoneal metastatic tumor cells by characterizing their genomic features through whole-exome sequencing.

We observed a unique base substitution signature biased toward C-to-A transversions in GC-derived malignant ascites. Interestingly, mutations detected from liver cirrhosis-derived benign ascites also presented predominant C-to-A transversions. Accordingly, these data may suggest that unknown mutagenic processes within ascitic fluid might promote C-to-A conversion. However, the result should be interpreted with caution, because the average VAF of the benign ascites-derived mutations detected was as low as ~9.3%. Although the mutation-calling software MuTect can detect mutations with VAFs as low as 10% and below with high sensitivity [29], it is required to confirm our result using a large sample set whether the biased C-to-A substitution is a characteristic of ascites-derived mutations.

The number and VAF of mutations, and tumor clonality showed a trend of correlation with the elapsed time between gastrectomy and the development of peritoneal carcinomatosis, and the overall survival duration after peritoneal carcinomatosis. However, given the low tumor purity, especially in primary tumors, we cannot rule out the possibility that the correlation pattern is false positive. Therefore, validation with a large sample set is required to claim that time course of clinical events such as the development of peritoneal carcinomatosis and overall survival can be estimated by mutation profiles.

Whole-exome sequencing revealed several candidate mutations in GC peritoneal carcinomatosis: mutations in druggable genes (*TEP1*, *PRKCD*, *BRAF*, *ERBB4*, *PIK3CA*, *HDAC9*, *FYN*, *FASN*, *BIRC2*, *FLT3*, *ROCK1*, *CD22*, and *PIK3C2B*) and mutations in metastasis-associated genes (*PTPN13*, *TNFSF12*, *L1CAM*, *DIAPH3*, *ROCK1*, *TGFBR1*, *MYO9B*, *NR4A1*, and *RHOA*). We also identified recurrent mutations in *COL4A6*, *INTS2*, and *PTPN13*. The *COL4A6* nonsense mutation (G543*) may result in the loss-of-function of COL4A6 because the mutation truncates ~68% of the COL4A6 protein. The functional loss of COL4A6, a type IV collagen found in the basement membrane, could disrupt the cell-matrix interaction and enhance cell invasion and metastasis [30, 31]. PTPN13 was reported to inhibit metastasis by dephosphorylation of Src or Her2 [32]. Our study provides a preliminary result, but validation experiments are required to prove whether the recurrent mutations frequently occur in malignant ascites. Therefore, the mutations should be validated with high detection sensitivity using high quality samples. Interestingly, by searching for the TCGA mutation data, we found a hepatocellular carcinoma patient harboring the *INTS2* recurrent mutation (G906K).

We found somatic mutations in the Rho-ROCK pathway genes, including *RHOA*, *ROCK1*, *ROCK2*, *FYN*, and *MYO9B*. Our gene ontology analysis revealed that the Rho-ROCK pathway-associated functional terms, including actin cytoskeleton, focal adhesion, and the Rho-protein signaling, were significantly overrepresented in the mutated genes in malignant ascites (Figure 3). A recent study demonstrated that the overexpression of mutant RhoA harboring an L57V mutation resulted in evasion of cell detachment-induced apoptosis, referred to as anoikis [13]. Since the capability for anchorage-independent growth and resistance against anoikis may be a prerequisite for the survival of tumor cells during metastasis, this *RHOA* mutation may provide a selective advantage for metastasis. We identified a nonsense mutation in *ROCK1* that introduces a premature stop codon at amino acid 285. This mutation may cause the loss-of-function of ROCK1, because most of the functional domains, including the Rho-binding domain, are truncated by the mutation. Given that treatment with the ROCK inhibitor Y-27632 prevents anoikis [13], tumor cells harboring the *ROCK1* nonsense mutation might be resistant to anoikis through the impairment of the Rho-ROCK pathway, similar to tumor cells harboring the RHOA L57V mutation.

We identified somatic mutations in *TGFBR1* and *NR4A1*, which are associated with the TGF-β pathway. Contrary to a tumor suppressive role in early-stage cancer, the TGF-β pathway contributes to tumor invasion and metastasis through epithelial-mesenchymal transition (EMT) in advanced cancer [33]. A recent study reported that NR4A1 activated the TGF-β-induced EMT and promoted metastasis through AXIN2- RNF12/ARKADIA-induced SMAD7 degradation [26]. Therefore, further studies are required whether the *NR4A1* mutation participates in the development of peritoneal carcinomatosis.

Our study revealed the genetic alterations and their clinical associations in GC peritoneal carcinomatosis through whole-exome sequencing of malignant ascites. However, there are several limitations, including the small sample size, the relatively low sequencing depth, low tumor purity of primary tumors, and a lack of functional validation. In the future, deep sequencing with a large sample set and subsequent functional studies would support our results, facilitating the discovery of therapeutic targets against GC peritoneal carcinomatosis.

## MATERIALS AND METHODS

### Sample collection

Surgical specimens of primary gastric tumors and their matched normal gastric tissues (non-malignant) were harvested and fresh frozen in Yonsei Cancer Center (Seoul, Korea). The normal gastric tissues used in this study were harvested from gastric regions at a distance from the primary tumor and exhibited no evidence of metaplasia and dysplasia. Matched malignant ascites were collected through paracentesis or catheter drainage of GC patients, whereas non-malignant ascites were obtained from patients with liver cirrhosis. The clinicopathologic characteristics were retrospectively reviewed based on the electronic medical records of the patients. Pathological diagnosis and staging were conducted according to the American Joint Committee on Cancer 7th staging system. This study was performed with the approval of the institutional review board.

### Whole-exome sequencing and data analysis

Genomic DNA was isolated from 24 tissues using the Puregene™ DNA purification kit (Qiagen, Venlo, Netherlands). Library construction and targeted exome enrichment were performed using the Illumina TruSeq DNA Sample Prep Kit (San Diego, CA, USA) and the SeqCap EZ Human Exome Library v2.0 kit (Roche NimbleGen, WI, USA), respectively. Next, paired-end sequencing was performed on the Illumina HiSeq 2000 sequencing instrument, according to the manufacturer's instructions, yielding ~100 bp-sized short sequencing reads. The sequencing reads were aligned on human reference genome 19 using Burrows Wheelers Aligner [34], and duplicates reads were removed using Picard (Broad Institute). Then, the remaining reads were calibrated and realigned using Genome Analysis Toolkit [35]. The realigned BAM files were analyzed using MuTect [29] and Strelka [36] to detect somatic single-nucleotide variants and insertions/deletions, respectively. Coding variants were selected by dbNSFP annotation [37] and germline variants were filtered out by dbSNP database (dbSNP version 132). SciClone was used to infer tumor clonality [38], and ASCAT v2.1 was used to estimate tumor purity [39]. We run all these programs under the default parameter settings.

### Data access

Our exome sequencing raw files are available from the NCBI SRA (http://www.ncbi.nlm.nih.gov/sra/) via accession number SRP043661.

## SUPPLEMENTARY MATERIALS TABLES AND FIGURES





## Abbreviations

GC: gastric cancer
VAF: variant allele frequency
