# Supplementary material for: Genetic alterations and their clinical implications in gastric cancer peritoneal carcinomatosis revealed by whole-exome sequencing of malignant ascites
Source: Oncotarget. 2016 Jan 22;7(7):8055–66. doi: 10.18632/oncotarget.6977 (PMC4884975; doi:10.18632/oncotarget.6977)
Supplement: Supplementary file 1 [file oncotarget-07-8055-s001.pdf]

Genetic alterations and their clinical implications in gastric cancer peritoneal carcinomatosis revealed by whole-exome sequencing of malignant ascites

Supplementary Materials

Supplementary Table S1: Somatic single-nucleotide variants identified in primary tumors and malignant ascites from eight GC patients

Supplementary Table S2: Insertion/deletions identified in primary tumors and malignant ascites from eight GC patients

Sample	comparison	Chrom	Position	REF	ALT	N_ref_count	N_var_count	T_ref_count	T_var_count	Gene	Function
P1	N_vs_T	1	152193501	ACTGCCCT	A	84	0	74	8	HRNR:NM_001009931:exon3:c.597_603del:p.S199fs,	frameshift deletion
P1	N_vs_A	1	152193501	ACTGCCCT	A	84	0	52	4	HRNR:NM_001009931:exon3:c.597_603del:p.S199fs,	frameshift deletion
P1	N_vs_A	8	10467670	TCTC	T	114	1	149	45	RP1L1:NM_178857:exon4:c.3935_3937del:p.1312_1313del,	nonframeshift deletion
P1	N_vs_A	8	10467680	TTCCTTC	T	106	0	152	41	RP1L1:NM_178857:exon4:c.3922_3927del:p.1308_1309del,	nonframeshift deletion
P1	N_vs_A	9	72003318	TGAG	T	21	0	14	10	FAM189A2:NM_004816:exon10:c.1102_1104del:p.368_368del,FAM189A2:NM_001127608:exon10:c.1102_1104del:p.368_368del,	nonframeshift deletion
P1	N_vs_A	15	79058940	GCGC	G	26	0	6	7	ADAMTS7:NM_014272:exon19:c.3310_3312del:p.1104_1104del,	nonframeshift deletion
P1	N_vs_A	19	20307915	TAA	T	68	0	38	27	ZNF486:NM_052852:exon4:c.397_398del:p.K133fs,	frameshift deletion
P1	N_vs_A	19	36002406	GCCACTGCTGCTGCCA	G	35	0	43	10	DMKN:NM_001126058:exon5:c.810_824del:p.270_275del,DMKN:NM_033317:exon5:c.810_824del:p.270_275del,DMKN:NM_001126057:exon5:c.810_824del:p.270_275del,DMKN:NM_001190349:exon5:c.810_824del:p.270_275del,DMKN:NM_001190348:exon5:c.810_824del:p.270_275del,	nonframeshift deletion
P1	N_vs_A	19	51532685	CCA	C	21	0	12	19	KLK12:NM_019598:exon6:c.618_619del:p.C206fs,KLK12:NM_145894:exon6:c.618_619del:p.C206fs,	frameshift deletion
P1	N_vs_A	19	58419932	T	TC	44	0	38	12	ZNF417:NM_001297734:exon3:c.1710dupG:p.R571fs,ZNF417:NM_152475:exon3:c.1713dupG:p.R572fs,	frameshift insertion
P1	N_vs_A	21	31964910	TTGGCTATGGAGGCCTGGACTG	T	146	0	147	56	KRTAP6-3:NM_181605:exon1:c.147_167del:p.49_56del,	nonframeshift deletion

P2	N_vs_T	17	39262108	CTCCAGCTGCTGCCGCCCCTCTTGCTGTGAA	C	94	0	53	6	KRTAP4-9:NM_001146041:exon1:c.469_498del:p.157_166del,	nonframeshift deletion
P2	N_vs_T	17	40839791	A	AGG	114	0	49	6	CNTNAP1:NM_003632:exon8:c.1098_1099insGG:p.G366fs,	frameshift insertion
P2	N_vs_A	10	103900543	A	AGGCAGCAACGCC	31	0	33	12	PPRC1:NM_015062:exon5:c.2278_2279insGGCAGCAACGCC:p.R760delinsRQQRR,PPRC1:NM_001288728:exon5:c.1918_1919insGGCAGCAACGCC:p.R640delinsRQQRR,PPRC1:NM_001288727:exon5:c.2278_2279insGGCAGCAACGCC:p.R760delinsRQQRR,	nonframeshift insertion
P4	N_vs_A	5	140763866	GA	G	25	0	17	11	PCDHGA7:NM_032087:exon1:c.1401delA:p.R467fs,PCDHGA7:NM_018920:exon1:c.1401delA:p.R467fs,	frameshift deletion
P4	N_vs_A	14	23531382	CT	C	26	0	37	15	ACIN1:NM_001164816:exon9:c.1086delA:p.Q362fs,ACIN1:NM_001164815:exon15:c.3147delA:p.Q1049fs,ACIN1:NM_001164817:exon10:c.993delA:p.Q331fs,ACIN1:NM_014977:exon16:c.3267delA:p.Q1089fs,ACIN1:NM_001164814:exon16:c.3228delA:p.Q1076fs,	frameshift deletion
P4	N_vs_A	14	92439181	ATTTAACAAAAAG	A	25	0	15	9	TRIP11:NM_004239:exon20:c.5587_5598del:p.1863_1866del,	nonframeshift deletion
P7	N_vs_A	4	87749180	G	GA	52	0	4	3	SLC10A6:NM_197965:exon4:c.726dupT:p.L243fs,	frameshift insertion
P7	N_vs_A	12	66707894	CTTAAA	C	177	0	20	5	HELB:NM_033647:exon5:c.1810_1814del:p.L604fs,	frameshift deletion

Supplementary Table S3: Somatic mutations identified in benign ascites from three liver cirrhosis patients

Sample	Symbol	Chr	Pos	Ref_allele	Var_allele	t_ref_count	t_var_count	VAF	n_ref_count	n_var_count	ref_aa	var_aa	aa_pos
N1	OBSCN	chr1	228467731	G	T	11	3	21.43	37	0	G	C	2536
N1	SNED1	chr2	241969887	G	A	41	4	8.89	64	1	V	I	134
N1	ADAMTS12	chr5	33549452	C	A	16	3	15.79	35	0	E	X	1388
N1	TCERG1	chr5	145858082	G	T	29	3	9.38	63	1	R	L	543
N1	PILRA	chr7	99971918	A	G	40	5	11.11	67	1	K	E	106
N1	C10orf140	chr10	21806630	G	T	16	3	15.79	36	1	P	Q	41
N1	EHD1	chr11	64627529	G	T	45	4	8.16	99	1	P	Q	261
N1	ARRB1	chr11	74994491	C	A	45	4	8.16	64	1	R	L	65
N1	UBE4A	chr11	118255604	C	A	23	3	11.54	59	1	R	S	786
N1	LRP10	chr14	23345977	C	A	42	4	8.7	65	1	P	Q	403
N3	SSR2	chr1	155981667	G	T	33	4	10.81	9	0	P	Q	56
N3	IGFN1	chr1	201182623	C	A	33	4	10.81	8	0	H	N	2868
N3	LBR	chr1	225607467	C	A	80	5	5.88	17	0	G	W	134
N3	NPAS2	chr2	101564802	C	A	55	5	8.33	9	0	P	H	146
N3	ITIH3	chr3	52830836	G	T	77	6	7.23	21	0	Q	H	121

N3	DDX60L	chr4	169340487	C	A	80	5	5.88	8	0	W	L	859
N3	SLC26A2	chr5	149357618	C	A	140	6	4.11	22	0	Q	K	135
N3	SVIL	chr10	29779789	C	T	54	5	8.47	10	0	M	I	967
N3	BTAF1	chr10	93742468	C	A	72	5	6.49	9	0	Q	K	725
N3	YLPM1	chr14	75276670	C	A	43	4	8.51	13	0	P	Q	960
N3	IFT140	chr16	1636194	C	A	89	6	6.32	30	0	E	D	364
N3	NPIPP1	chr16	15198362	G	T	87	6	6.45	21	0	L	I	341
N3	CWC25	chr17	36963075	C	A	71	6	7.79	10	0	R	L	282
N3	LILRA2	chr19	55086954	A	C	31	4	11.43	10	0	Y	S	296
N3	ACRC	chrX	70824479	C	A	42	4	8.7	9	0	P	Q	451

Supplementary Table S4: Mutated druggable genes (DGIdb)

Sample	Gene	Drug name	Interaction Type	SciClone_Cluster
P1	BIRC6	AEG40826	antagonist	3
P1	BIRC6	TL 32711	antagonist	3
P1	BIRC6	GDC0917	antagonist	3
P1	BIRC6	LCL161	antagonist	3
P1	TEP1	GRN163L	inhibitor	1
P1	PRKCD	SOPHORETIN	inhibitor	1
P1	PRKCD	KAI-9803	inhibitor	1
P4	TEP1	GRN163L	inhibitor	3
P4	ERBB4	DACOMITINIB	inhibitor	3
P4	ERBB4	BMS-599626	inhibitor	3
P4	ERBB4	BMS-690514	inhibitor	3
P4	ERBB4	PELITINIB	inhibitor	3
P4	ERBB4	POZIOTINIB	inhibitor	3
P4	ERBB4	GEFITINIB	inhibitor	3
P4	ERBB4	CI-1033	inhibitor	3
P4	PIK3CA	BAY80-6946	inhibitor	3
P4	PIK3CA	INK-1117	inhibitor	3
P4	PIK3CA	GDC-0032	inhibitor	3
P4	PIK3CA	BYL719	inhibitor	3
P4	PIK3CA	BEZ235	inhibitor	3
P4	PIK3CA	BGJ398	inhibitor	3
P4	PIK3CA	BKM120	inhibitor	3
P4	PIK3CA	GDC-0941	inhibitor	3
P4	PIK3CA	GDC-0980	inhibitor	3
P4	PIK3CA	GSK2636771	inhibitor	3
P4	PIK3CA	PF-4691502	inhibitor	3
P4	PIK3CA	PI-103	inhibitor	3
P4	PIK3CA	PKI-587	inhibitor	3
P4	PIK3CA	PWT33597	inhibitor	3
P4	PIK3CA	PX-866	inhibitor	3
P4	PIK3CA	SF1126	inhibitor	3

P4	PIK3CA	XL147	inhibitor	3
P4	PIK3CA	XL-765	inhibitor	3
P4	PIK3CA	SOPHORETIN	inhibitor	3
P4	PIK3CA	BUPARLISIB HYDROCHLORIDE	inhibitor	3
P4	PIK3CA	DACTOLISIB	inhibitor	3
P4	PIK3CA	GSK2126458	inhibitor	3
P4	PIK3CA	SAR260301	inhibitor	3
P4	PIK3CA	XL765	inhibitor	3
P4	PIK3CA	ON01910	inhibitor	3
P4	PIK3CA	NVP-BGT226	inhibitor	3
P4	PIK3CA	PF-04691502	inhibitor	3
P4	FYN	VX-680	inhibitor	5
P4	FYN	DASATINIB	inhibitor	5
P4	HDAC9	VORINOSTAT	inhibitor	3
P4	HDAC9	BELINOSTAT	inhibitor	3
P4	HDAC9	PIVANEX	inhibitor	3
P4	HDAC9	VALPROIC ACID	inhibitor	3
P4	HDAC9	SODIUM PHENYLBUTYRATE	inhibitor	3
P4	HDAC9	GIVINOSTAT	inhibitor	3
P4	HDAC9	SB939	inhibitor	3
P4	HDAC9	CUDC-101	inhibitor	3
P4	HDAC9	PCI-24781	inhibitor	3
P4	HDAC9	DACINOSTAT	inhibitor	3
P4	HDAC9	PANOBINOSTAT	inhibitor	3
P4	BRAF	ARQ736	inhibitor	3
P4	BRAF	XL281	inhibitor	3
P4	BRAF	LGX818	inhibitor	3
P4	BRAF	REGORAFENIB	inhibitor	3
P4	BRAF	SORAFENIB	inhibitor	3
P4	BRAF	RO5212054	inhibitor	3
P4	BRAF	RAF265	inhibitor	3
P4	BRAF	VEMURAFENIB	inhibitor	3
P4	BRAF	DABRAFENIB	inhibitor	3
P4	BRAF	GSK2118436	inhibitor	3
P4	BRAF	DABRAFENIB MESYLATE	inhibitor	3
P4	BRAF	1096708-71-2	inhibitor	3
P4	BRAF	SORAFENIB TOSYLATE	inhibitor	3
P4	BRAF	DASATINIB	inhibitor	3
P4	BRAF	TEMSIROLIMUS	inhibitor	3
P4	BRAF	CHIR-265	inhibitor	3
P4	BRAF	PLX3603	inhibitor	3
P4	BRAF	ZELBORAF	inhibitor	3
P4	BRAF	R7204	inhibitor	3
P4	BRAF	BMS-908662	inhibitor	3
P6	FASN	ORLISTAT	inhibitor	3

P6	FASN	CERULENIN	inhibitor	3
P8	BIRC2	GDC0917	antagonist	4
P8	BIRC2	AT-406	antagonist	4
P8	BIRC2	TL 32711	antagonist	4
P8	BIRC2	LCL161	antagonist	4
P8	BIRC2	AEG40826	antagonist	4
P8	CD22	EPRATUZUMAB	antibody	4
P8	CD22	ANTI-CD22	antibody	4
P8	CD22	MOXETUMOMAB PASUDOTOX	antibody	4
P8	PIK3C2B	BAY80-6946	inhibitor	5
P8	PIK3C2B	BEZ235	inhibitor	5
P8	PIK3C2B	BGJ398	inhibitor	5
P8	PIK3C2B	BKM120	inhibitor	5
P8	PIK3C2B	BYL719	inhibitor	5
P8	PIK3C2B	GDC-0941	inhibitor	5
P8	PIK3C2B	GDC-0980	inhibitor	5
P8	PIK3C2B	GSK2636771	inhibitor	5
P8	PIK3C2B	PF-4691502	inhibitor	5
P8	PIK3C2B	PI-103	inhibitor	5
P8	PIK3C2B	PKI-587	inhibitor	5
P8	PIK3C2B	PWT33597	inhibitor	5
P8	PIK3C2B	PX-866	inhibitor	5
P8	PIK3C2B	SF1126	inhibitor	5
P8	PIK3C2B	XL147	inhibitor	5
P8	PIK3C2B	XL-765	inhibitor	5
P8	PIK3C2B	SOPHORETIN	inhibitor	5
P8	FLT3	CABOZANTINIB	inhibitor	6
P8	FLT3	SUNITINIB	inhibitor	6
P8	FLT3	PLX3397	inhibitor	6
P8	FLT3	MIDOSTAURIN	inhibitor	6
P8	FLT3	AMUVATINIB	inhibitor	6
P8	FLT3	TANDUTINIB	inhibitor	6
P8	FLT3	ENMD-0276	inhibitor	6
P8	FLT3	LESTAURTINIB	inhibitor	6
P8	FLT3	AS703569	inhibitor	6
P8	FLT3	QUIZARTINIB	inhibitor	6
P8	FLT3	PONATINIB	inhibitor	6
P8	FLT3	BORTEZOMIB	inhibitor	6
P8	FLT3	CLOFARABINE	inhibitor	6
P8	FLT3	4'-N-BENZOYLSTAUROSPORINE	inhibitor	6
P8	FLT3	IDARUBICINE	inhibitor	6
P8	FLT3	SORAFENIB TOSYLATE	inhibitor	6
P8	FLT3	CRENOLANIB	inhibitor	6
P8	FLT3	AZACITIDINE	inhibitor	6
P8	FLT3	PLX108-01	inhibitor	6

P8	FLT3	SORAFENIB	inhibitor	6
P8	FLT3	XL999	inhibitor	6
P8	FLT3	SUNITINIB MALATE	inhibitor	6
P8	FLT3	SU5614	inhibitor	6
P8	FLT3	4SC-203	inhibitor	6
P8	FLT3	SORAFENIB	antagonist	6
P8	ROCK1	SAR407899	inhibitor	7
P8	ROCK1	CETHRIN	inhibitor	7
P8	ROCK1	RHO-KINASE INHIBITOR	inhibitor	7

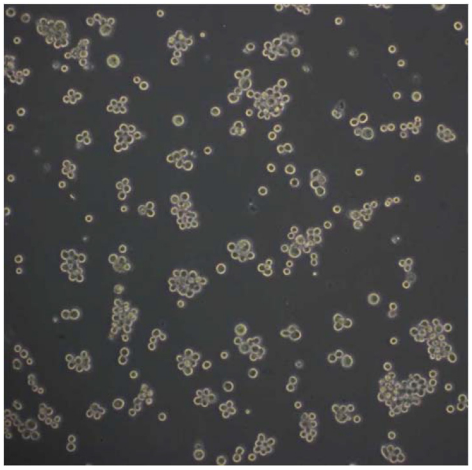

Supplementary Figure S1: Cytological examination of gastric cancer cells derived from malignant ascites.

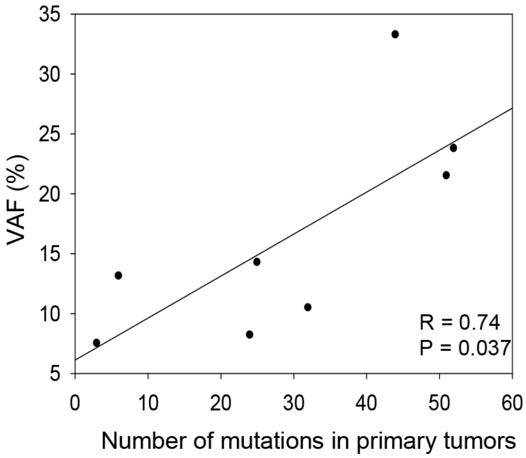

Supplementary Figure S2: Correlation between the number of mutations and VAF.

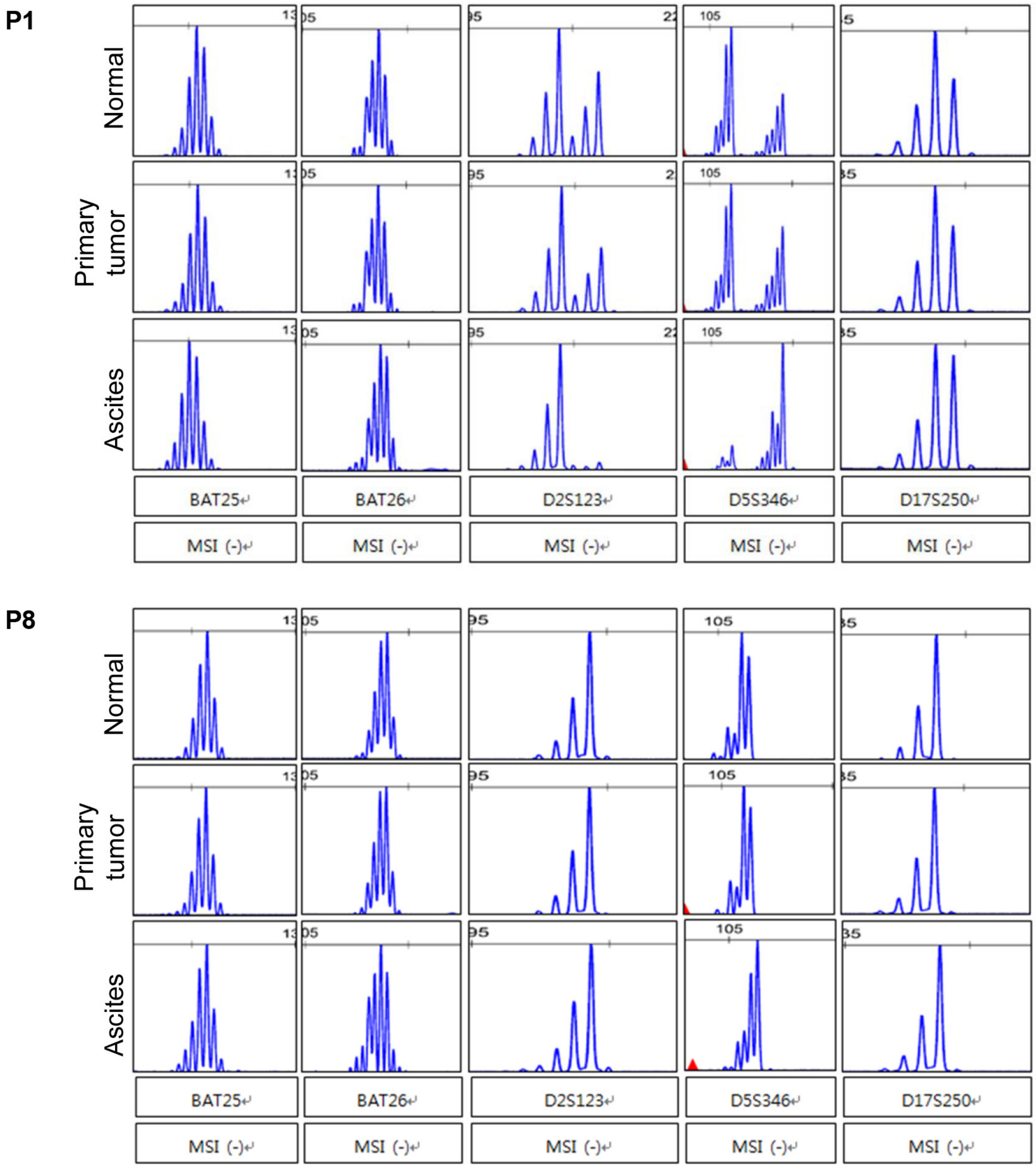

Supplementary Figure S3: A microsatellite instability test for hypermutated tumor samples (P1 and P8).

Base substitutions at tri-nucleotide context

G[C->T]G	G[C->T]C	G[C->T]A	G[C->T]T	G[C->A]G	G[C->A]C	G[C->A]A	G[C->A]T	G[C->G]G	G[C->G]C	G[C->G]A	G[C->G]T
C[C->T]G	C[C->T]C	C[C->T]A	C[C->T]T	C[C->A]G	C[C->A]C	C[C->A]A	C[C->A]T	C[C->G]G	C[C->G]C	C[C->G]A	C[C->G]T
A[C->T]G	A[C->T]C	A[C->T]A	A[C->T]T	A[C->A]G	A[C->A]C	A[C->A]A	A[C->A]T	A[C->G]G	A[C->G]C	A[C->G]A	A[C->G]T
T[C->T]G	T[C->T]C	T[C->T]A	T[C->T]T	T[C->A]G	T[C->A]C	T[C->A]A	T[C->A]T	T[C->G]G	T[C->G]C	T[C->G]A	T[C->G]T
G[A->G]G	G[A->G]C	G[A->G]A	G[A->G]T	G[A->C]G	G[A->C]C	G[A->C]A	G[A->C]T	G[A->T]G	G[A->T]C	G[A->T]A	G[A->T]T
C[A->G]G	C[A->G]C	C[A->G]A	C[A->G]T	C[A->C]G	C[A->C]C	C[A->C]A	C[A->C]T	C[A->T]G	C[A->T]C	C[A->T]A	C[A->T]T
A[A->G]G	A[A->G]C	A[A->G]A	A[A->G]T	A[A->C]G	A[A->C]C	A[A->C]A	A[A->C]T	A[A->T]G	A[A->T]C	A[A->T]A	A[A->T]T
T[A->G]G	T[A->G]C	T[A->G]A	T[A->G]T	T[A->C]G	T[A->C]C	T[A->C]A	T[A->C]T	T[A->T]G	T[A->T]C	T[A->T]A	T[A->T]T

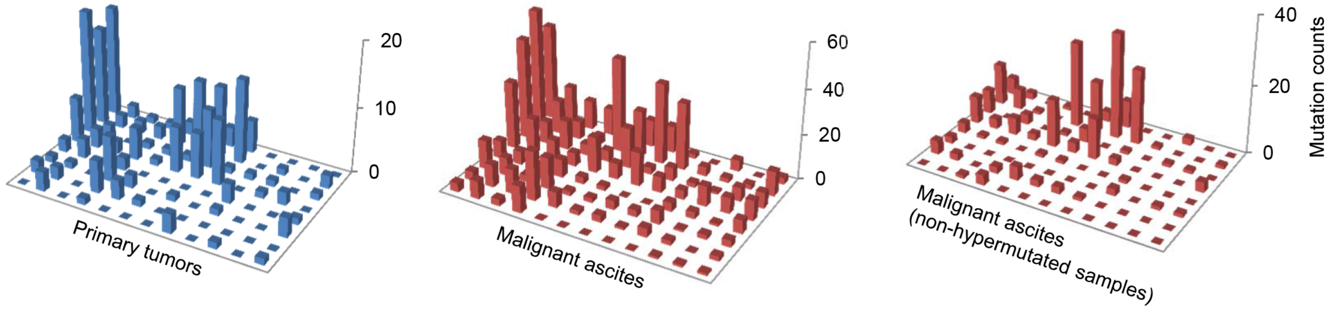

Supplementary Figure S4: 96 mutational base substitutions in tri-nucleotide contexts. The patterns of mutational base substitutions found in all malignant ascites (middle) and non-hypermutated malignant ascites (right) were presented separately.

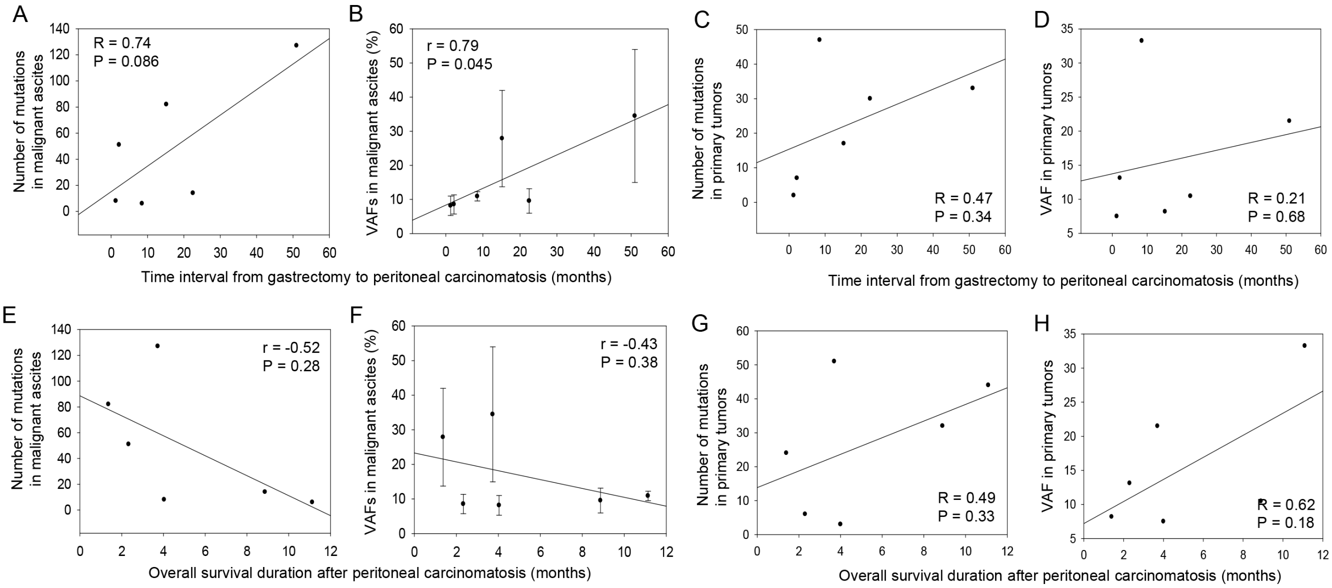

Supplementary Figure S5: (A) The correlation between the number of mutations in malignant ascites and the time interval from gastrectomy to the diagnosis of peritoneal carcinomatosis. (**B**) The correlation between the VAFs of mutations in malignant ascites and the time interval from gastrectomy to the diagnosis of peritoneal carcinomatosis. (**C**) The correlation between the number of mutations in primary tumors and the time interval from gastrectomy to the diagnosis of peritoneal carcinomatosis. (**D**) The correlation between the VAFs of mutations in primary tumors and the time interval from gastrectomy to the diagnosis of peritoneal carcinomatosis. (**E**) The correlation between the number of mutations in malignant ascites and the overall survival duration after the diagnosis of peritoneal carcinomatosis. (**F**) The correlation between the VAFs of mutations in malignant ascites and the overall survival duration after the diagnosis of peritoneal carcinomatosis. (**G**) The correlation between the number of mutations in primary tumors and the overall survival duration after the diagnosis of peritoneal carcinomatosis. (**H**) The correlation between the VAFs of mutations in primary tumors and the overall survival duration after the diagnosis of peritoneal carcinomatosis.

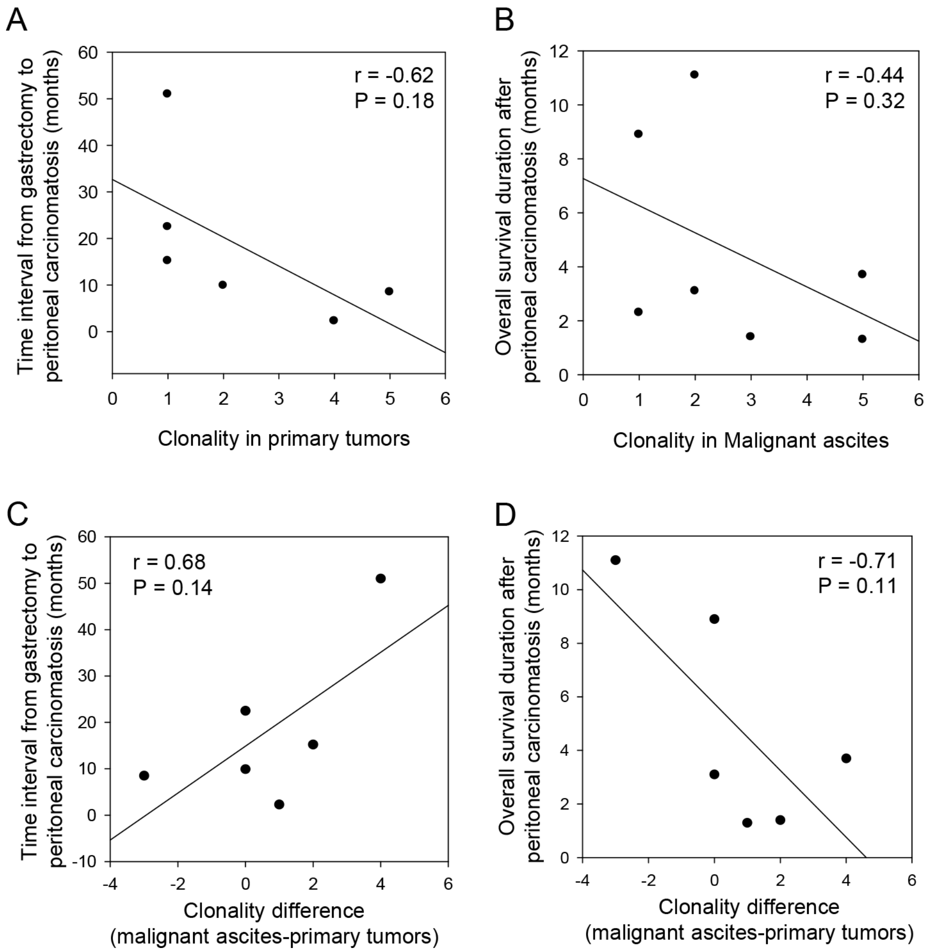

Supplementary Figure S6: (A) The correlation between clonality in primary tumors and the time interval from gastrectomy to the diagnosis of peritoneal carcinomatosis. (**B**) The correlation between clonality in malignant ascites and the overall survival duration after the diagnosis of peritoneal carcinomatosis. (**C**) The correlation between clonality differences during metastasis and the time interval from gastrectomy to the diagnosis of peritoneal carcinomatosis. (**D**) The correlation between clonality differences and the overall survival duration after the diagnosis of peritoneal carcinomatosis.

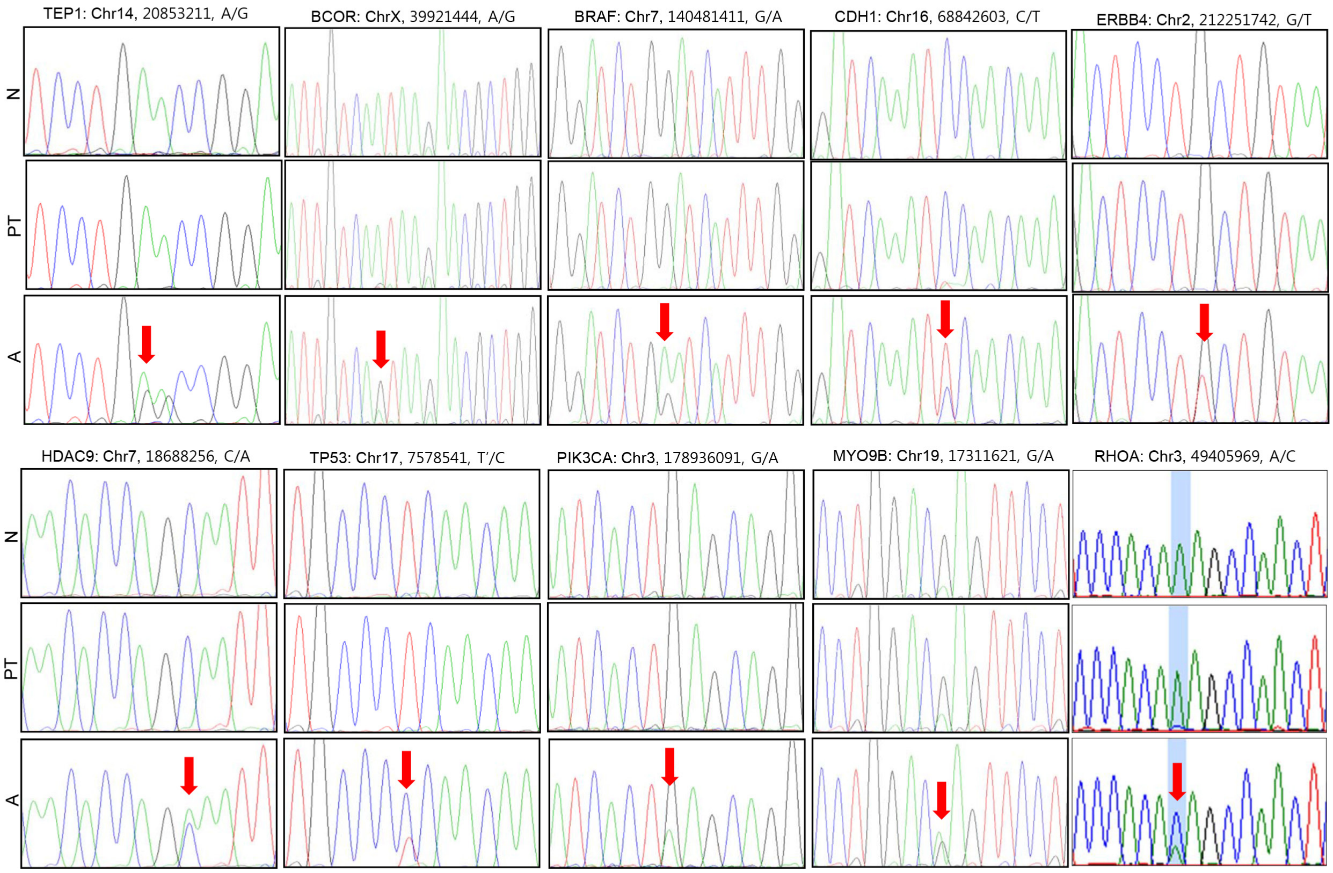

Supplementary Figure S7: Validation of detected mutations by Sanger sequencing.

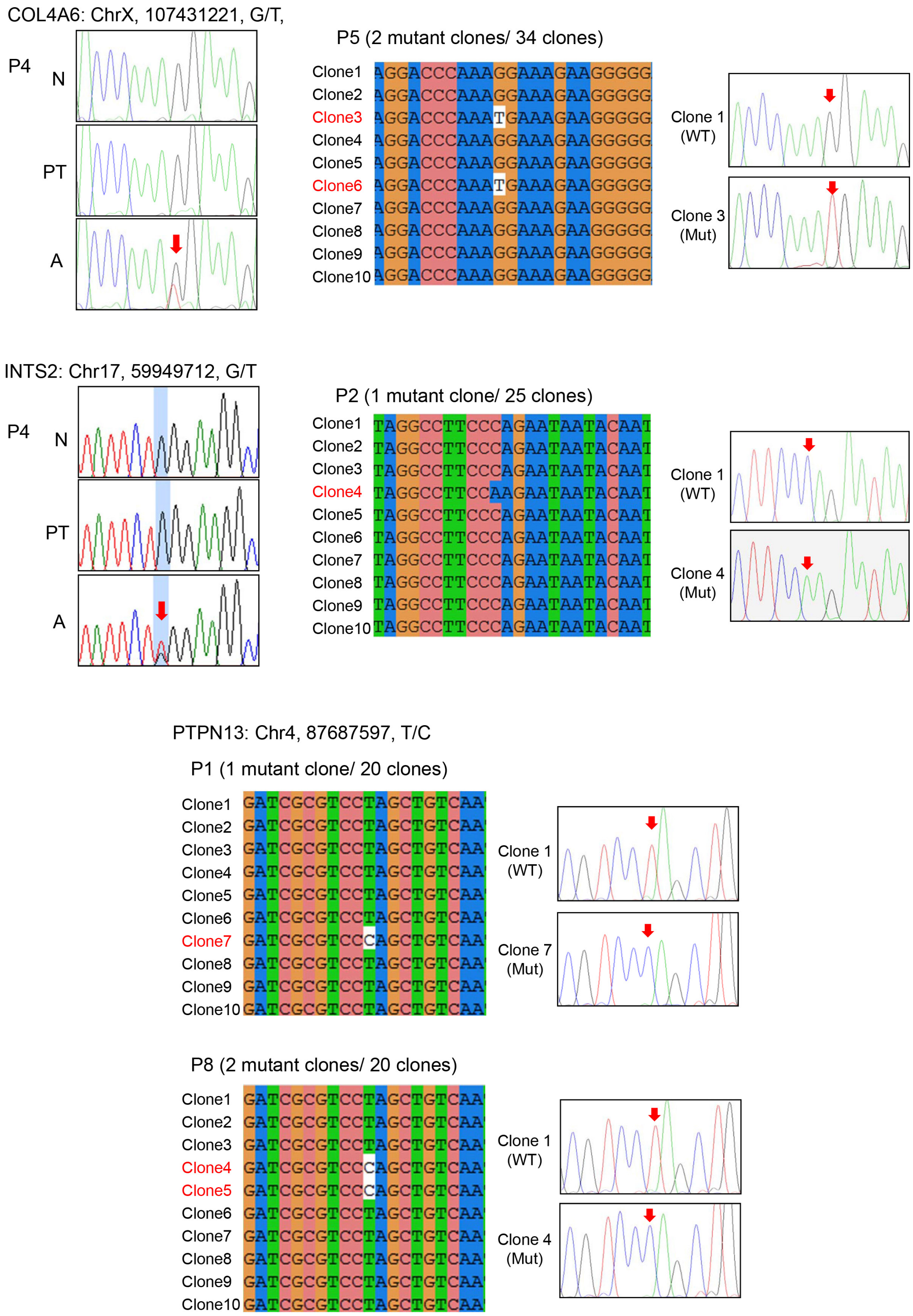

Supplementary Figure S8: Validation of three recurrent mutations by Sanger sequencing. For mutations with low VAFs below detection limit of Sanger sequencing, we cloned PCR fragments harboring recurrent mutations into vector and sequenced each clone by Sanger sequencing. The number of mutated and sequenced clones is indicated.
